# Diagnosis and treatment of right ventricular metastasis from esophageal squamous cell carcinoma: An unusual case report and a literature review

**DOI:** 10.1097/MD.0000000000036404

**Published:** 2023-12-08

**Authors:** Lingyun Luo, Xuelian Luo

**Affiliations:** a Tongji Hospital, Tongji Medical College, Huazhong University of Science and Technology, Wuhan, Hubei, China; b Department of Oncology, The Third Affiliated Hospital of Chongqing Medical University, Chongqing, China.

**Keywords:** early diagnosis, intracardiac metastasis, squamous cell esophageal cancer, treatment

## Abstract

**Background:**

Right ventricular metastasis from esophageal squamous cell carcinoma is very rare and only seen in few case reports. Patients with cardiac metastasis have a poor prognosis with a median survival period of 4 weeks due to the lack of standardized and effective treatment guidelines. Therefore, we aimed to clarify the feature and treatment of cardiac metastasis through literature review and reporting of an unusual case.

**Case:**

We reported a case of a 67 years-old man diagnosed as right ventricular metastasis from esophageal squamous cell with the help of echocardiography and pathological biopsy. Moreover, the patient survival period reached an astonishing 6 months, which far exceeded 4 weeks reported in previous literature.

**Methods:**

We searched for relevant literature in the past decade on PUBMED and summarized the content of the literature to better clarify cardiac metastasis.

**Conclusion:**

Cardiac metastatic likely to occur in the elderly and in the right side of heart which related to hemodynamics. Surgical resection of metastatic tumors is the main treatment method, but patients usually die during the perioperative period due to its complexity and difficulty. Meanwhile, we have proposed some potentially effective treatment measures.

## 1. Introduction

The incidence of cardiac metastatic tumors is up to 40 times higher than that of primary tumors.^[1]^ What is more, cardiac metastatic tumors are often asymptomatic that they are found only at autopsy and seldom be reported.^[2]^ Mainly malignant melanoma and primary mediastinal tumors, but also lymphoma, lung carcinoma, breast, esophagus, and leukemia, are cause of cardiac metastases.^[3]^ In case of cardiac metastatic tumors, the prognosis is poor and therapeutic options limited.^[4]^ Generally, patients with symptomatic heart metastases die within a few weeks, with a median survival of 4 weeks.^[5–7]^ Malignant tumors can metastasize to the heart through 3 pathways: direct infiltration; blood diffusion; lymphatic spread. Direct invasion of mediastinum and intrathoracic tumors often leads to pericardial metastasis; Blood dissemination usually causes myocardial metastasis; Lymphatic spread often causes pericardial metastasis. Endocardial metastasis is relatively rare, usually caused by blood invading tumor cells entering the ventricular cavity, and can also be secondary to myocardial metastasis.^[8]^ Tumors spread can occur, in descending order, to the pericardium, myocardium, epicardium, endocardium and less frequently to the intracavitary regions with predominance to the right-side of the heart.^[9]^ We report a case of right ventricular metastasis from esophageal squamous cell carcinoma with relatively good prognosis. Presently, there is a lack of standard treatment methods for cardiac metastatic tumors. According to literature reports, the therapy is mainly targeted at the treatment of metastasis tumor, such as undergoing cardiac metastatic tumor resection. Surgery can relieve obstruction and alleviate patient symptoms, as well as diagnosis the nature of cardiac tumors. The patient we reported got relief after the early surgery and chemotherapy. Unfortunately, the COVID-19 broke and the patient died on Dec 19th, 2022. This patient took about 6 months from the diagnosis of cardiac metastasis to death, which far exceeded the median survival period of 4 weeks reported in previous literature. Considering the rarity of cardiac metastases and the current lack of effective treatment measures, we will report on the patient diagnosis and treatment process and share our treatment measures in the hope of providing valuable insights for the treatment of cardiac metastases.

### Timeline

**Figure 1.**
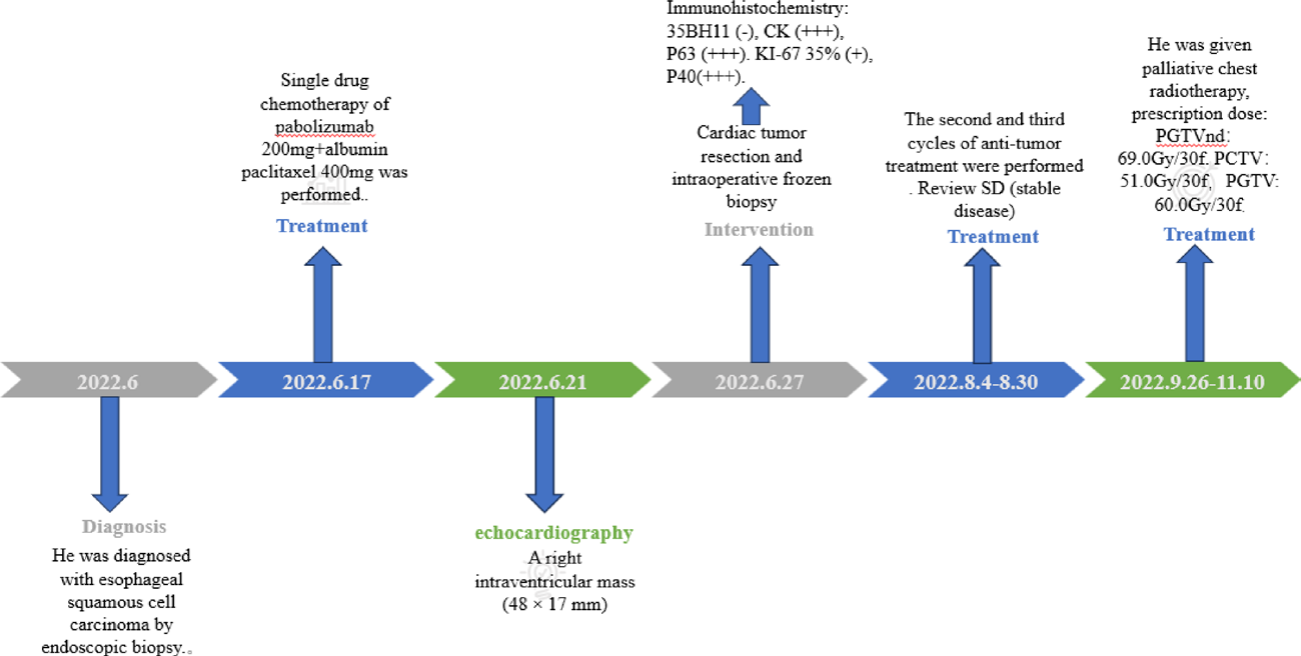
The important milestones related to our diagnoses and interventions.

The important milestones related to our diagnoses and interventions are depleted (Fig. 1).

## 2. Case report

**Figure 2.**
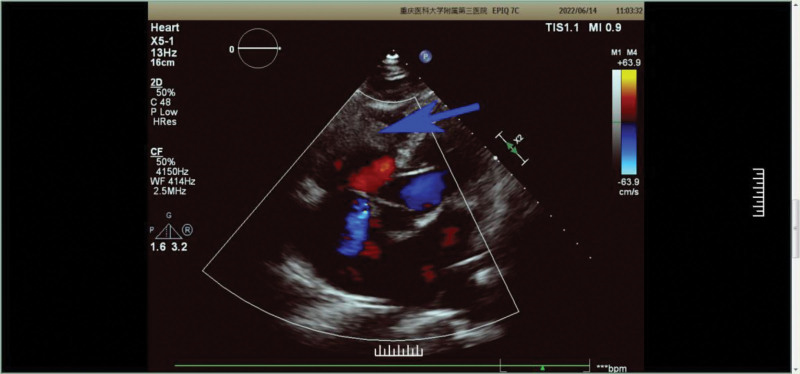
Echocardiography on June 14th: the location indicated by the blue arrow indicates the possibility of a tumor occupying lesion.

**Figure 3.**
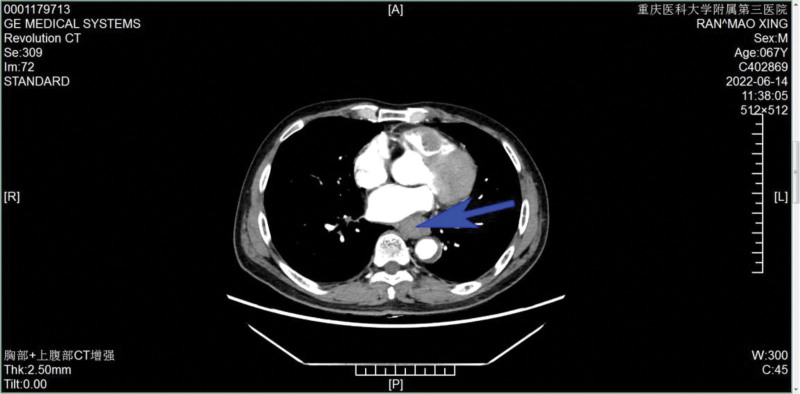
The thoracic computed tomography on June 14th: the location indicated by the blue arrow indicates the possibility of a tumor occupying lesion.

A 67 years-old Tujia nationality man was admitted to our institution in June 2022 with a history of dysphagia, slight chest pain when obstruction, and dyspnea when climbing stairs or going uphill. Gastroscope showed that a new organism which was 29 to 35 cm away from the esophagus could be seen protruding from the lumen, with rigid wall, central ulceration, covered with filthy moss, brittle texture, and easy to bleed when touched. Then he was diagnosed with esophageal squamous cell carcinoma by endoscopic biopsy. On June 14th, echocardiography revealed the presence of an intracardiac mass inside the right ventricle (Fig. 2). The echocardiography showed there is a slight increase in echogenicity in the right ventricle, and considering the possibility of an embolus based on medical history, a space occupying lesion needs to be excluded, further examination is recommended; widening of the aortic sinus and ascending part; mild symmetrical thickening of the left ventricular wall; mitral valve, tricuspid valve, aortic valve, pulmonary valve local regurgitation; left ventricular diastolic dysfunction. Then thoracic computed tomography was requested for a better assessment about the mass intracardiac (Fig. 3). The thoracic computed tomography results were as follows: Space occupying lesions in the middle and lower thoracic segments of the esophagus. Considering the possibility of esophageal cancer, multiple lymph nodes in the mediastinum are shown, follow-up is recommended. There are scattered solid and sub-solid nodules in both lungs, and metastatic tumors need to be excluded. It is recommended to follow up closely. Double emphysema; there is a little chronic inflammation in the medial segment of the middle lobe of the right lung, the lower lingual segment of the upper lobe of the left lung, and the lower lobe of both lungs. Right ventricular filling defect shadow, considering thrombosis, reexamination is recommended.

Overall, the mass was suspected to be tumor or embolus. Hypersensitive D dimer is 543 ng/mL and Color Doppler ultrasound of blood vessels in extremities showed no thrombus. We chose to treat esophageal cancer first after multi-department consultation and communication with the patient family. On June 17th, 2022, single drug chemotherapy of pabolizumab 200 mg + albumin paclitaxel 400 mg was performed successfully. After treatment, swallowing obstruction and chest pain slightly improved but the patient developed skin itching and rash.

**Figure 4.**
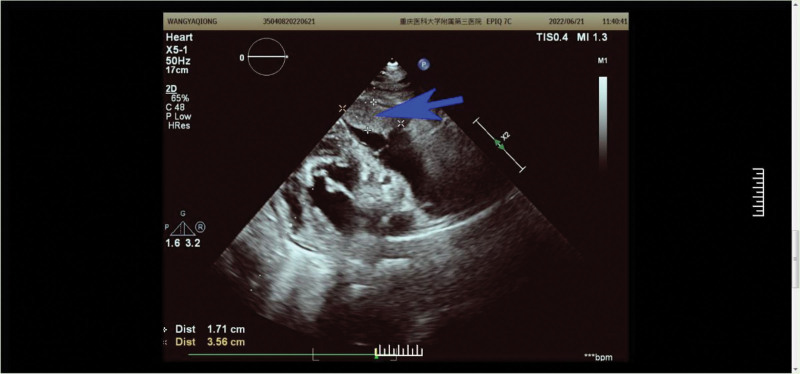
Review color ultrasound on June 21st: the location indicated by the blue arrow indicates a right intraventricular mass (48 × 17 mm).

On June 21st, Review color ultrasound showed a right intraventricular mass (48 × 17 mm) (Fig. 4), the boundary is clear, the shape is regular, and the base is wide. It swings with the heart beating. Part of the echo located in the outflow tract of the right ventricle, and part of the outflow tract is about 10.3 mm wide. After the consultation of cardiac surgery in our hospital, the patient underwent cardiac tumor resection and intraoperative frozen biopsy on June 27, 2022. The surgical process is as follows: after successful tracheal intubation under general anesthesia, the patient is placed in a flat position, with a jugular vein catheter, a radial artery catheter, and a urinary catheter. After routine iodine disinfection, the patient is covered with a cloth. The sternum is incised in the middle of the sternum, and the skin is cut subcutaneously. The sternum is longitudinally sawn, bone wax is used to stop bleeding, the sternum is opened, and a pericardium is cut. The patient is suspended, and the ascending aorta is intubated, the superior and inferior vena cava is intubated, and the left atrium is intubated. Extracorporeal circulation is established routinely. After cardiac arrest, cut open the right atrium and conduct intracardiac exploration: The tumor is located on the anterior wall of the right ventricle, widely distributed from the apex to the distal end of the right ventricular outflow tract. The surface of the tumor is uneven, crispy, and the base is wide. The pulmonary artery trunk is blocked, and the tumor is gradually removed. A small amount of tumor tissue is taken and sent for intraoperative frozen pathological examination. The heart cavity is repeatedly rinsed with ice and saline, and the pulmonary artery blocking forceps are removed. No tumor tissue is found in the pulmonary artery during exploration. The intraoperative frozen pathological report is “metastatic malignant tumor.” The tricuspid valve was well closed during the right ventricular injection test, and the right atrial incision was sutured. After rewarming, the ascending aorta was opened. The heart was defibrillated once and the cardiopulmonary bypass was paralleled, gradually stopping the extracorporeal circulation. After removing the extracorporeal circulation, a temporary cardiac pacing lead was placed on the surface of the right ventricle, and a pericardial mediastinal drainage tube was placed to completely stop bleeding. After counting the number of pairs, the chest closure surgery was completed layer by layer. Return to the CCU ward safely.

**Figure 5.**
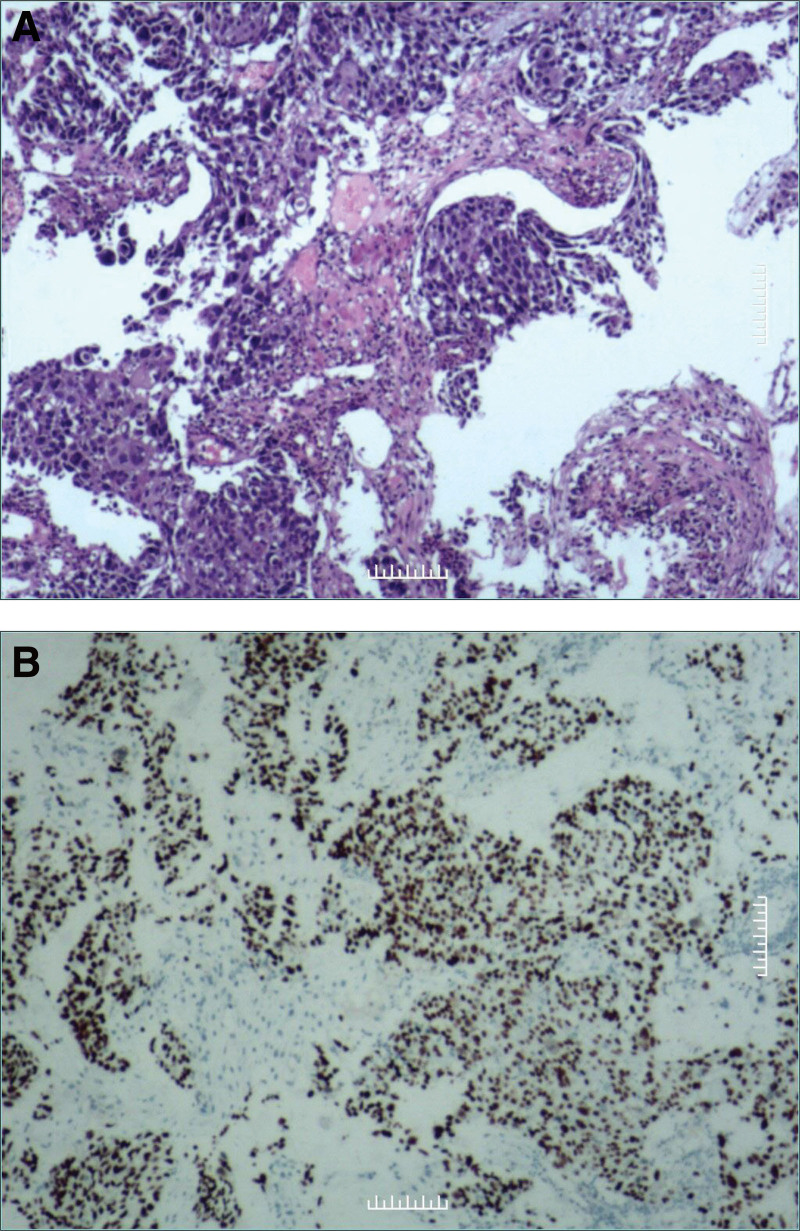
Pathological findings from the resected specimen on June 27th. (A) Cancer cells are invading into the heart muscle (Hematoxylin and eosin staining). (B) The resected specimen is positive to CK and p40. CK is a marker of tumors from epithelial tissue and p40 is a marker of squamous cell carcinoma.

Pathological examination (frozen section) on June 27, 2022 was reported: Cardiac space-occupying, malignant tumor with massive necrosis, consistent with the metastasis of poorly differentiated squamous cell carcinoma, it is recommended to examine the esophagus. Immunohistochemistry: 35BH11 (−), CK (+++), P63 (+++). KI-67 35% (+), P40 (+++) (Fig. 5). Besides that, the reason the mass has been considered as an esophagus metastasis is that intracardiac lesions from esophagus cancer are described in the literature.^[3]^

**Figure 6.**
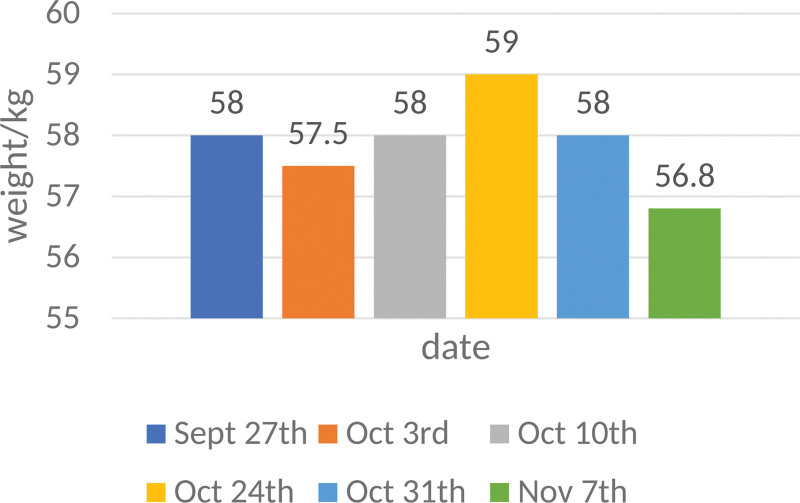
Patient weight change from Sept 27th to Nov 7th.

**Figure 7.**
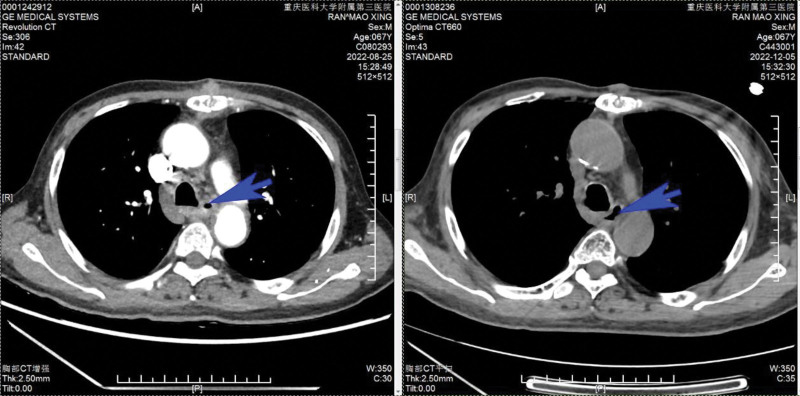
In this picture, the area indicated by the arrow is the esophagus. The left one is the CT on August 25th and the right one is the CT on December 5th. We can find that the thickening of the middle and lower esophageal wall is significantly reduced compared to before, and the stricture of esophagus is relieved.

The second and third cycles of anti-tumor treatment were performed on August 4, 2022 and August 30, 2022. Review SD (stable disease). From September 26, 2022 to November 10, 2022, he was given palliative chest radiotherapy, prescription dose: PGTVnd: 69.0 Gy/30f. PCTV: 51.0 Gy/30f, PGTV: 60.0 Gy/30f. The patient maintained a stable weight during radiotherapy (Fig. 6). By the computed tomography and enhanced CT, it can be seen that the treatment in the 3 months has significantly improved the patient condition (Fig. 7).

It is pity that, due to the outbreak of COVID-19, the patient eventually suffered from severe pulmonary infection on Dec 19th. The infection rapidly progressed and worsened under the influence of various inflammatory factors. And the patient was died of the acute respiratory distress syndrome 2 weeks later.

## 3. Discussion

In this report, the authors mainly describe a case of right ventricular metastasis from Esophageal Squamous Cell. The patient ‘s condition was well controlled after cardiac tumor resection and postoperative periodic chemoradiotherapy. The patient survival period reached 6 months, which is a significant improvement compared to the 4 weeks median survival reported in the previous literature. We used “Metastatic cardiac tumors [Title/Abstract]” as search term in PUBMED and filter the article type as case report, the publication data as 10 years, 24 case reports were found. Three articles which were misdiagnosed as cardiac metastases were excluded. Finally, the data from the remaining 21 articles is summarized in the table below. Hereafter, we will present characteristics of MCT in 4 parts.

### 3.1. Literature review

From the Table 1, we can draw the following 3 points. Firstly, MCT likely to occur in the elderly. Secondly, MCT usually transferred to the right side of heart. We speculate that this may be related to hemodynamics, as cardiac metastasis occurs through blood flow, and the blood flow velocity in the right heart is slower than that in the left heart, making it more suitable for tumor tissue colonization. Finally, in clinical practice, resection of the primary tumor is commonly used as a treatment for cardiac metastases, but most patients usually die during the perioperative period because of the difficulty and challenge of surgery.

**Table 1:** Literature review of metastatic cardiac tumor.

Authors	Publication years	Case number	Age (yr)/sex	Location of tumor	Histopathological examination	Tumor size (cm^2^ or cm^3^)	Treatment	Prognosis (mo)
Angela Kornberger et al^[10]^	2017	1	69/M	interventricular septum.	HCC	7.2X4.5X5.6	Surgery	5.5/Death
María Facenda-Lorenzo et al^[11]^	2017	1	83/M	RV and RA	HCC	6.0X3.0	Unknown	Unknown
Yoshihiko Onishi et al^[12]^	2023	1	71/F	RA	RCC	Unknown	Surgery	9/Alive
Ryo Yamaguchi et al^[13]^	2021	2	62/F;49/M	LA	EC	Unknown	Surgery	Unknown
Yoshio Sudo et al^[14]^	2013	1	70/F	Right ventricular outflow tract	CC	Unknown	Surgery	2/Alive
Raunak M Nair et al^[15]^	2019	1	68/F	Left side of the heart	VC	2.5X1.1	Unknown	Unknown
Seddiq M et al^[16]^	2022	1	14/F	RA	HL	4.7 × 4.7 × 5	Chemotherapy	24/Alive
Gupta D et al^[17]^	2016	1	2/F	LV	Neuroblastoma	1.75 × 1.91	chemotherapy1.25/Death	Unknown
Maebayashi A et al^[18]^	2020	1	49/F	RV and RA	UL	4.9 × 2.6	Surgery	Unknown
Pallangyo P et al^[19]^	2022	1	67/M	RV and RA	SCLC	Unknown	dual chamber pacemaker implantation	18/Death
Osati E et al^[20]^	2017	1	21/M	RV	osteosarcoma	5.99 × 5.59	Chemothearapy	Unknown
Kalvakuri K et al^[21]^	2016	1	49/F	RV	CSCC	8 × 4 × 2.5	Surgery	0.2/Death
Hajsadeghi S et al^[22]^	2020	1	60/F	RV and RA	UCA	7.5X4X3	Surgery	0.008/Death
Zakhartseva LM et al^[23]^	2018	1	24/M	RV	Testicular tumor	Unknown	Surgery	Unknown
Kawakami J et al^[24]^	2022	1	71/F	Right interatrial septum	Liposarcoma	Unknown	Provision of palliative care	Unknown
Tregubenko P et al^[25]^	2021	1	63/M	RV	LSCC	4.6X3.6	Palliative radiotherapy	0.5/Death
Mahalwar G et al^[26]^	2022	1	29/M	RV	TGCT	7.4X6.0	Do-not-resuscitate comfort care	0/Death
Goldberg AB et al^[27]^	2019	1	56/F	RA	Chondrosarcoma	5.8X2.9	Surgery	Unknown
Tian L et al^[28]^	2022	1	48/M	Right atrioventricular groove	SCLC	3.6X5.4	Chemothearapy and pericardiocentesis	5/Death
Burazor I et al^[9]^	2018	3	50/F	RV	Melanoma	3.5X4X4.5	Surgery and chemotherapy	60/Alive
			77/F	septum and apex of heart	LBSCC	Unknown	Chemotherapy	9/Death
			70/F	RA	PDAC	6X6X3	Palliative therapy	0.25/Death
Tsujii Y et al^[29]^	2017	1	76/F	RV	Colon cancer	5.4X3.2	Chemotherapy	24/alive
Total		24	54.08/9M and 15F	20 right side and 4 left side				11.25

CC = colon cancer, CSCC = cervical squamous cell carcinoma, EC = esophageal cancer, HCC = hepatocellular carcinoma, HL = Hodgkin lymphoma, Uterine leiomyosarcoma, LA = left atrium, LBSCC = lung basaloid squamous cell carcinoma, unknown means we could not find any related information in the literature, LSCC = laryngeal squamous cell carcinoma, LV = left ventricle, PDAC = poorly differentiated adenocarcinoma of the colon, RA = right atrium, RCC = renal cell carcinoma, RV = right ventricle, SCLC = small cell lung cancer, TGCT = testicular germ cell tumor, UCA = uterine cervical adenocarcinoma, VC = vulvar cancer.

### 3.2. Generalities

Several papers have shown that tumors of the heart are more often metastatic than primary.^[30,31]^ Once the primary tumors have metastasized to the heart, the prognosis is extremely poor, with death usually occurring in less than a year. In the context of this case, it also shows the importance of early diagnosis and early treatment in improving survival and prognosis.^[7]^ Most tumors of the heart are metastases from melanoma, lung or esophageal cancer, breast cancer. As most are asymptomatic, cardiac metastases are often detected or confirmed at autopsy,^[32]^ which is particularly important for doctors to be aware of. And doctors can diagnose cardiac metastases with the help of medical imagery such as CT, echocardiography and MRI^[33]^ and pathological biopsies.

### 3.3. Treatment

Although there is currently a lack of standard treatment methods for cardiac metastases, we combined this case report with previous literature to summarize some treatment measures which may be helpful in prolonging the median survival of patients. On the one hand, we need treatment for cardiac metastatic lesions, including: resection of cardiac metastatic tumors: surgery can relieve obstruction and alleviate patient symptoms, and also clarify the nature of cardiac tumors; local radiotherapy: Radiotherapy can be performed on cardiac metastases, but the sensitivity of cardiac metastases to radiotherapy is poor and the prognosis is poor; systemic chemotherapy: Systemic chemotherapy can improve patient survival. On the other hand, we need to treat the complications caused by cardiac metastasis. For metastatic cardiac tumors usually cause a wide spectrum of common cardiac symptoms and signs. Therefore, only by properly managing the complications of cardiac metastases can the survival period of patients be effectively extended.

### 3.4. Reflection

Although the patient in this case unfortunately developed a severe lung infection and SIRS as a result of the novel coronavirus infection and eventually died of MODS, the prompt diagnosis and standardized treatment process by the doctors at the Third Affiliated Hospital of CQMU is to be commended. On reflection, the authors think that clinical doctors, when encountering similar cases, should make a precise diagnosis with the help of medical imagery such as CT, echocardiography and MRI and pathological biopsies and take early treatment. In this way, not only we can fulfill the responsibilities of being doctors, but also the patients can get relief and have a better prognosis. We hope that this paper can provide some suggestions for the treatment of cardiac metastases in clinical practice in order to improve the prognosis of patients and extend their median survival.

## 4. Conclusion

We comprehensively discussed the characteristics, diagnosis, and treatment of cardiac metastases through literature review and reporting a case. In a nutshell, Firstly, MCT likely to occur in the elderly and usually transferred to the right side of heart. Additionally, resection of the primary tumor is commonly used as a treatment for cardiac metastases, but most patients usually die during the perioperative period because of the difficulty and challenge of surgery. More future work is needed to improve the life quality and extended median survival of the patients with MCT.

## 5. Limitations

This paper focuses on a case report and literature review to discuss cardiac metastasis. The level of evidence is obviously not comparable to clinical studies such as randomized controlled trial.

## Author contributions

**Conceptualization:** Xuelian Luo.

**Data curation:** Xuelian Luo.

**Formal analysis:** Xuelian Luo.

**Funding acquisition:** Xuelian Luo.

**Investigation:** Xuelian Luo.

**Methodology:** Xuelian Luo.

**Project administration:** Xuelian Luo.

**Resources:** Lingyun Luo.

**Software:** Lingyun Luo.

**Supervision:** Lingyun Luo.

**Validation:** Lingyun Luo.

**Visualization:** Lingyun Luo.

**Writing – original draft:** Lingyun Luo.

**Writing – review & editing:** Lingyun Luo.
